# Influence of a pre-stimulation with chronic low-dose UVB on stress response mechanisms in human skin fibroblasts

**DOI:** 10.1371/journal.pone.0173740

**Published:** 2017-03-16

**Authors:** Marie-Catherine Drigeard Desgarnier, Frédéric Fournier, Arnaud Droit, Patrick J. Rochette

**Affiliations:** 1 Axe Médecine Régénératrice, Centre de Recherche du CHU de Québec – Université Laval, Hôpital du Saint-Sacrement, Québec, Quebec, Canada; 2 Centre de Recherche en Organogénèse Expérimentale de l'Université Laval/LOEX, Université Laval, Québec, Quebec, Canada; 3 Centre de Protéomique, Centre de Recherche du CHU de Québec – Université Laval, Québec, Quebec, Canada; 4 Département de Médicine Moléculaire, Université Laval, Québec, Canada; 5 Département d’Ophtalmologie et ORL - Chirurgie Cervico-Faciale, Université Laval, Québec, Canada; University of Alabama at Birmingham, UNITED STATES

## Abstract

Exposure to solar ultraviolet type B (UVB), through the induction of cyclobutane pyrimidine dimer (CPD), is the major risk factor for cutaneous cancer. Cells respond to UV-induced CPD by triggering the DNA damage response (DDR) responsible for signaling DNA repair, programmed cell death and cell cycle arrest. Underlying mechanisms implicated in the DDR have been extensively studied using single acute UVB irradiation. However, little is known concerning the consequences of chronic low-dose of UVB (CLUV) on the DDR. Thus, we have investigated the effect of a CLUV pre-stimulation on the different stress response pathways. We found that CLUV pre-stimulation enhances CPD repair capacity and leads to a cell cycle delay but leave residual unrepaired CPD. We further analyzed the consequence of the CLUV regimen on general gene and protein expression. We found that CLUV treatment influences biological processes related to the response to stress at the transcriptomic and proteomic levels. This overview study represents the first demonstration that human cells respond to chronic UV irradiation by modulating their genotoxic stress response mechanisms.

## Introduction

Skin cancers represent the most frequent type of cancer [1]. Exposure to solar ultraviolet (UV), through the induction of pre-mutagenic DNA lesions, is the major risk factor for cutaneous cancer development [2]. More precisely, UVB (280–315 nm) are the most carcinogenic wavelengths reaching the Earth surface [3]. The two UVB-induced mutagenic DNA damage are the cyclobutane pyrimidine dimer (CPD) and the pyrimidine (6–4) pyrimidine photoproducts (6-4PP) [4]. If UV-induced DNA damage remain unrepaired, they can lead to UVB signature mutations found in skin cancer [5]. However, the main and most mutagenic UV-induced DNA damage is the CPD [4, 6, 7], which is responsible for C→T and CC→TT transition mutations found in skin cancer [8–12].

Even if UVB are the major contributor of skin cancer, they have also positive effects and applications. First, they are used in dermatology for phototherapy in order to treat different skin conditions [13]. They are also critical for vitamin D_3_ fixation [14, 15]. Also, in response to UVB, the skin neuroendocrine system responds differently with, among others, the stimulation of corticotropin-releasing factor (CRF) expression [16].

In human cells, UVB-induced DNA damage stimulate various molecular mechanisms to prevent the conversion of pre-mutagenic lesions such as the CPD into cancer driver mutations. These mechanisms signal the DNA damage to the cell, and then mediate DNA lesions removal or their tolerance [17]. When the decision is made to remove the lesion, the DNA damage response (DDR) is activated to either restore DNA by the nucleotide excision repair (NER) or to safely discard the damaged cell by programmed cell death [17, 18]. An early mechanism involved in CPD repair is the activation of DNA damage checkpoint that activates cell cycle delay to allow efficient repair. The regulation of those mechanisms is important to avoid mutagenicity. NER pathway is particularly important to prevent mutagenesis and is a critical mechanism for UVB cancer prevention. Indeed, patient deficient in the NER pathway (*Xeroderma Pigmentosum*; XP) have 1 000-fold increase of UV-induced skin cancer [19]. The protection mechanisms against UV-induced genotoxic stress have been extensively studied after subjecting cells or animal models with single acute UVB irradiation reviewed in [17, 20]. However, little is known about how those mechanisms are influenced by chronic exposure to UVB light. More precisely, the potential adaptive response of cells after chronic UVB irradiation is poorly understood [21, 22]. Knowing that we are physiologically exposed to repeated chronic low dose of UV (CLUV), it becomes crucial and relevant to understand how molecular mechanisms respond to recurrent irradiations.

In many studies, it has been found that pre-treating cells with chronic low amount of mutagenic agents lead to an adaptive cellular response render the cells more resistant to a subsequent stress against mutagenic agents [21–24]. However, most studies focused on adaptive response with non-physiological agents [21, 23, 25]. For example, one of the first studies demonstrating an adaptive response in human cells has shown that pre-stimulating dermal fibroblasts with chronic low doses of quinacrine mustard enhances CPD repair [23]. Notwithstanding, the effect of a physiological exposure to a CLUV dose has been studied [26, 27]. However, the effect of a CLUV treatment on DNA repair efficiency is somehow controversial. Indeed, it has been reported in mouse skin, that a CLUV dose decreases DNA repair capacity [26] with CPD accumulation and persistence [28, 29]. On the other hand, it has been revealed that CLUV treatment can lead to a faster DNA repair in skin type IV [30].

Although those studies have reported an effect of the CLUV treatment on DNA repair of UV-induced CPD, the consequences of this CLUV treatment and how cells respond after subsequent UVB dose, particularly on the DDR pathway has not been studied. Here, we have investigated whether an exposure of human cells to a CLUV treatment induces some changes at different stress response level. More precisely, we have determined the effect of a CLUV treatment of normal human dermal fibroblasts (NHDF) on the cellular response mechanisms to genotoxic stress, including CPD repair rate, cell cycle arrest and sensitivity to UV-induced apoptosis. In this study, NHDF were used as a model of human non-transformed cell strains and UVB were used as the most mutagenic UV wavelengths reaching the Earth surface. This study does not aim to evaluate each pathway in detail but rather offers an overview of the genotoxic stress response modulated by a chronic UV irradiation. Our data revealed that a CLUV treatment induces persistent residual CPD. Furthermore, our results show that CLUV treatment enhances the repair of newly formed CPD, delay cell cycle progression, but does not sensitize cells to apoptosis. Taken together, our results demonstrate that NHDF cells are able to modulate their DDR pathways following a chronic UVB pre-stimulation.

## Results

### 1. Effect of a CLUV pre-stimulation on DNA repair

**Fig 1:**
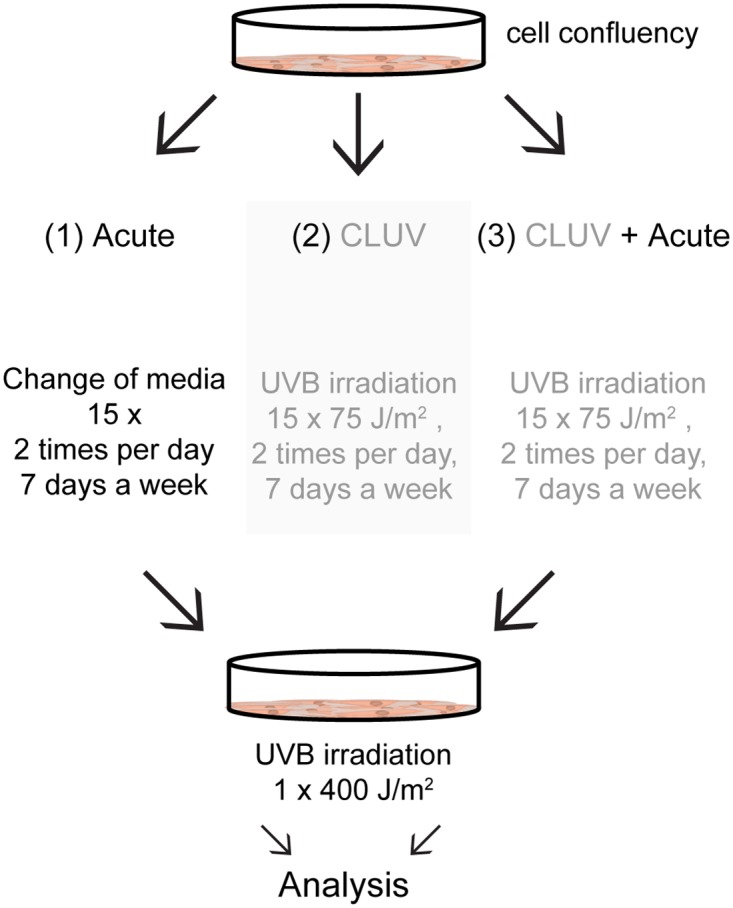
Schematic representation of the irradiation protocol. Confluent NHDF were irradiated with different UVB irradiation protocols. Three conditions were used: (1) single UVB dose, (2) CLUV treatment and (3) CLUV followed by a single UVB dose. (1) Acute treatment is a single UVB irradiation of 400 J/m^2^; (2) CLUV treatment consists UVB irradiations of 75 J/m^2^ every 12 h for 7.5 days (15 irradiations). (3) Cells are irradiated with the CLUV treatment described in (2) followed by the single UVB irradiation described in (1) 12 h after the last CLUV irradiation. Cells subjected to the different irradiation protocols were then used for further analysis.

**Fig 2:**
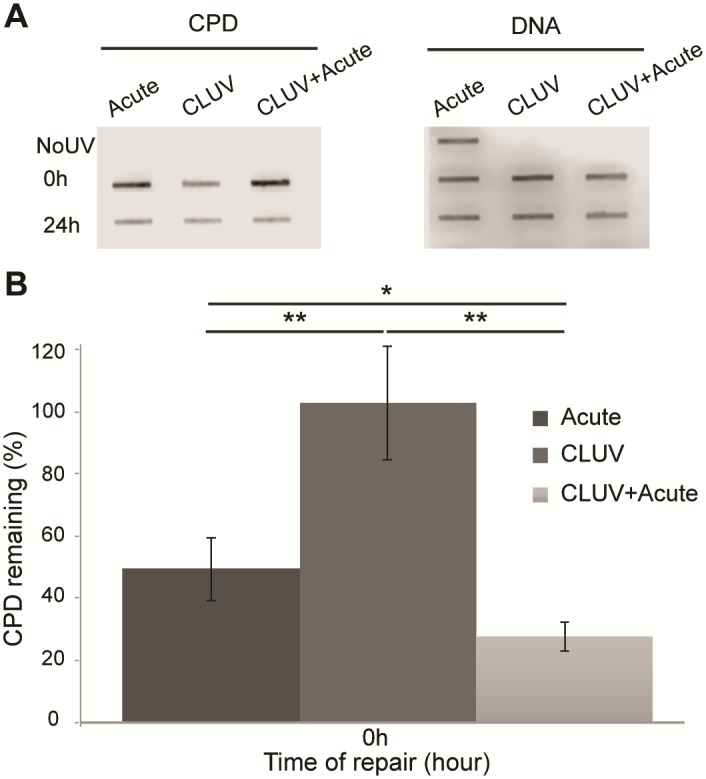
CPD repair rate is enhanced by the CLUV pre-stimulation in NHDF. **(A)** Immuno-slot-blot showing the level of CPD and DNA. NHDF were irradiated with a single UVB dose (Acute), a CLUV irradiation or a CLUV followed by a single acute UVB dose (CLUV+Acute), as described in Fig 1. Zero and 24 h post-irradiation, DNA was harvested and applied on a membrane. Revelation of CPD and DNA was performed using specific monoclonal antibodies. NoUV is used as a negative control and DNA as a loading control. **(B)** Representation of the quantity of CPD repair after different UVB treatments. Quantitative analysis of the immuno-slot-blot detecting CPD is performed by measuring the signal intensity at each time points post-UV and compare it to the 0 h for each UVB treatment condition (Acute, CLUV, CLUV+Acute). The signal at 0 h corresponds to 100% of the CPD signal for each UVB treatment independently. The results then describe the CPD removal relative to the initial CPD amount for each UVB treatment (Acute, CLUV and CLUV+Acute). Normalization is performed using the corresponding DNA signal as previously [31]. Results are presented as means ± SEM. *P-value* was evaluated using the student’s t-test (*p < 0.05; **p < 0.01). Experiment was performed using 3 strain cells (N = 3) at least in duplicate (n = 2).

We first aimed to determine the influence of CLUV pre-stimulation on UV-induced CPD repair. CPD were induced by an acute UVB treatment (400 J/m^2^) following or not a CLUV pre-stimulation (schematically represented in Fig 1). We first showed that the CLUV treatment induces CPD that still remain unrepaired 24 h post-irradiation (Fig 2A, left panel and Fig 2B). On the other hand, we can observe that a CLUV pre-stimulation enhance CPD repair (Fig 2B). More precisely, 72% of CPD are repaired 24 h post-irradiation in cells when they are pre-stimulated with a CLUV treatment where it reaches only 50.5% in non-pre-stimulated cells (*p <* 0.05). Moreover, since the CLUV treatment induces persistent CPD that remain in the genome 24 h post-irradiation, the repair rate derived in CLUV pre-treated cells take into account the newly formed CPD by the acute irradiation and the persistent CPD, thus the rate of newly formed CPD repair is underestimated (Fig 2A and 2B).

Cells subjected to the single UVB dose were removed from the incubator at the same frequency and length than the CLUV treated cells and the culture media was replaced at the frequency as well. This was done to ensure that the CLUV effect was not the result of the stress induced by the experimental procedure.

### 2. Consequence of a CLUV treatment on cell cycle

Previous studies have shown that under UV stress, cell cycle progression is halted to allow an effective DNA repair or to induce efficient apoptosis, thus preventing replication over mutagenic DNA damage [17, 32]. Indeed, previous analysis on human dermal fibroblasts demonstrated that a halt in cell cycle is required for effective UV-induced CPD repair [33]. Thereby, to determine the influence of a CLUV treatment on cell cycle progression, we have analyzed cell cycle using flow cytometry in CLUV treated cells and compared with acute UVB treated and un-irradiated cells. For this experiment, we used 200 J/m^2^ of UVB as acute dose to induced a similar amount of CPD as the residual CPD induced by the CLUV treatment. CPD are known to block cell cycle progression [32] and therefore, it was important to compare conditions (CLUV vs single acute UVB) with the same amount of CPD. As shown in Fig 2A, there are 2 times more CPD induced by the single acute UVB irradiation of 400 J/m^2^ than the residual CPD induced by the CLUV treatment. For the CLUV treatment, we used the protocol depicted in Fig 1.

**Fig 3:**
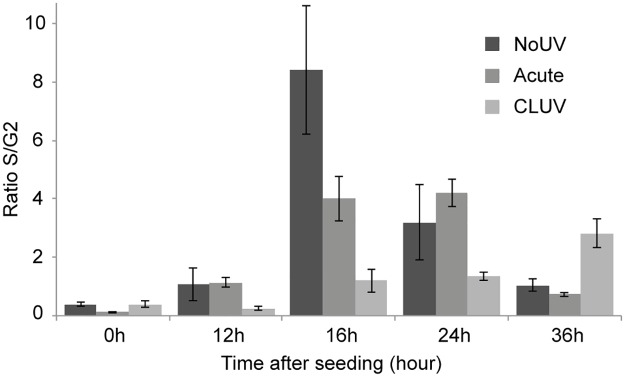
Cell division analysis after single UVB and CLUV irradiation. Cells were either irradiated with either a CLUV treatment (CLUV) or a single UVB dose of 200 J/m^2^ (Acute). Un-irradiated cells (NoUV) were used as control. Synchronization in G0 was achieved by keeping the cells at full confluency for 12 days and then re-seeded at low density (8.3x10^3^ cells/cm^2^). S-phase recovery derived as S/G2 ratio was assayed during 36 h using PI staining FACS analysis. Data are presented as means ± SEM using 4 independent cell strains (N = 4).

Cells were synchronized by keeping them at full confluency for 12 days and then re-seeded at low density to measure the S-phase recovery time. As shown in Fig 3, un-irradiated cells enter in S-phase 16h after their release, where it takes between 16 and 24 h for the acute irradiated cells. We can observe a S-phase initiation after 36 h in CLUV treated cells, but the recovery is still not completed at the longest time point analyzed (36 h). It is important to note that we have confirmed that the CLUV treated cells are not senescent and can replicate post-treatment (data not shown).

### 3. Consequence of a CLUV pre-stimulation on UVB-induced cell death

**Fig 4:**
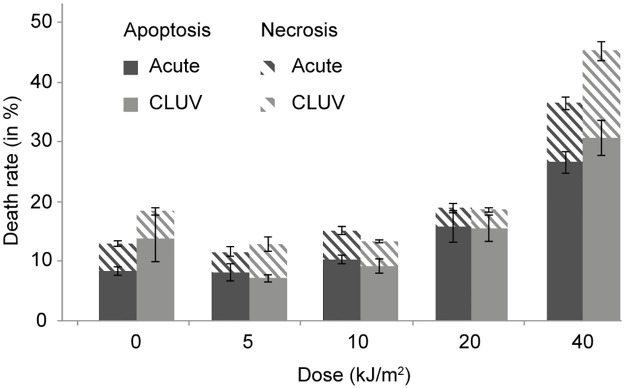
Effect of a CLUV pre-stimulation on UV-induced cell death sensitivity. NHDF CLUV pre-stimulated (CLUV) or not (Acute) were irradiated with UVB doses ranging from 0 to 40,000 J/m^2^. Sensitivity to UV-induced apoptosis and necrosis was assessed by FACS analysis, 16 h post-irradiation. Dashed bars represent the percentage of necrotic cells and solid bars are apoptotic cells. Data are expressed as means ± SEM of four independent NHDF cell strains (N = 4). Significance was evaluated using the student’s *t-test*.

It is well established that DNA damage trigger apoptosis [34, 35]. Indeed, various mechanisms take place after DNA damage induction to protect cells against the conversion of those mutagenic DNA damage into mutations. Programmed cell death is one of the most important protection mechanism against genotoxic stress [36]. Thus, to evaluate whether a CLUV pre-stimulation affects cell death, we examined cell sensitivity to UVB in CLUV pre-stimulated and un-stimulated cells (i.e. not receiving the CLUV pre-stimulation). We found no significant difference in UVB-induced cell death sensitivity in CLUV pre-stimulated cells when compared to un-stimulated cells (Fig 4). Indeed, at UVB doses ranging from 0 to 20,000 J/m^2^, the level of apoptotic and necrotic cells is virtually identical between CLUV pre-treated and un-stimulated cells. At the highest UVB dose use (40,000 J/m^2^), an increase in UV-induced necrosis and apoptosis sensitivity can be observed in CLUV pre-stimulated cells. However, this difference is not statistically significant (p = 0.08).

### 4. Microarray analysis of CLUV-induced transcriptomic changes

**Fig 5:**
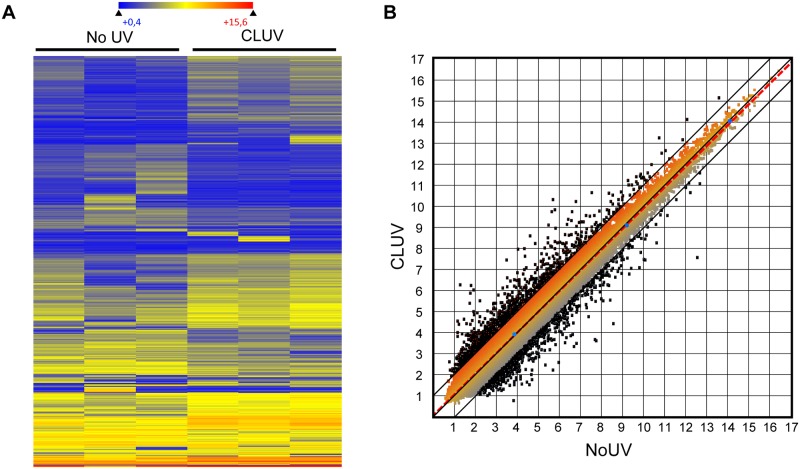
CLUV treatment induces transcriptomic changes. After NHDF were subjected or not to the CLUV treatment, total RNA was extracted to analyze gene profiling. For this experiment, the CLUV treatment is performed using 100 J/m^2^ of UVB instead of 75 J/m^2^. The experiment was performed in triplicate using 3 different NHDF strains. **(A)** Heatmap depicting the significantly deregulated genes in CLUV treated NHDF and un-irradiated controls. This experiment was performed in 3 different NHDF strains, and the heatmap clearly shows the reproducibility of CLUV-induced changes between strains. The color scale is based on the log2 expression level values. Hierarchical clustering was performed on rows based on the Euclidian distance. Genes indicated in dark blue correspond to those whose expression is very low, whereas highly expressed genes are shown in red. **(B)** Scatter plot of log2 signal intensity for 60 000 targets covering the entire human transcriptome. The signal for CLUV cells at 0 h (y-axis) is plotted against un-irradiated cells (Control, No UVB) (x-axis). All the >2-fold deregulated genes between the 2 conditions are represented by black dots. The 3 blue points are 3 controls (*B2M*, *TUBB*, *GOLGA1*). The transcription level of those genes is known to be stable, independent of cell type and condition [39].

It has been reported that stress induces gene expression changes [37] and particularly that UV induces the expression of genes involved in DDR response [38]. Thus, as the CLUV pre-stimulation influences DNA repair capacity, cell death sensitivity and cell cycle, we investigated the changes at the transcriptome level induced by this CLUV treatment. Therefore, a gene profiling analysis was performed to determine the entire human gene expression in CLUV pre-stimulated cells and in un-irradiated controls (Fig 5). The heat-map depicting all 2-fold deregulated genes for each replicate displays that the gene expression differences found between the CLUV pre-treated cells and the un-irradiated controls are reproducible (Fig 5A).

Scatter plot analysis comparing gene expression of CLUV pre-stimulated cells and un-irradiated cells highlight on up-regulated and down-regulated genes expression caused by the CLUV pre-treatment (Fig 5B). Microarray analysis underlines a total of 948 deregulated genes caused by the CLUV treatment. To evaluate correlation between gene expression patterns after CLUV treatment and their biological consequences, A BiNGO analysis was performed. A list of annotation was generated and classified according to biological processes that indicate different modulated cellular pathways significantly deregulated after a CLUV treatment (Table 1). Particularly, this induces important changes including some that are directly related to the stress response pathway (Table 1). More precisely, this comprises 151 deregulated genes implicated in the “response to stress” goID process (S1 Table). This represents 15.9% of all deregulated genes.

**Table 1:** List of biological processes deregulated by a CLUV treatment.

goID	Biological process	Number of up-regulated gene	Number of down-regulated gene	Number total of gene deregulated	Number total of gene in this process	*p-value*
65007	Biological Regulation	251	239	490	19974	8.1E-15
50789	Regulation of biological process	241	220	461	19036	5.4E-13
6950	Response to stress	78	73	151	5126	7.5E-08
48522	Positive regulation of cellular process	96	87	183	5537	4.8E-14
48518	Positive regulation of biological process	112	103	215	6956	1.4E-13
48519	Negative regulation of biological process	91	78	169	5414	1.2E-10
50794	Regulation of cellular process	229	201	430	18116	4.8E-10
48523	Negative regulation of cellular process	87	66	153	4955	2.6E-09
70995	NADPH oxidation	3	1	4	5	6.9E-07
48585	Negative regulation of response to stimulus	28	30	58	1533	1.2E-06
23051	Regulation of signaling	54	64	118	3605	1.2E-08
50896	Response to stimulus	150	157	307	12214	1.8E-08
31401	Positive regulation of protein modification process	22	30	52	1326	1.6E-06
48583	Regulation of response to stimulus	69	71	140	4560	6.8E-09
9966	Regulation of signal transduction	50	55	105	3178	1.5E-08
51247	Positive regulation of protein metabolic process	31	36	67	1769	1.7E-07
32270	Positive regulation of cellular protein metabolic process	29	33	62	1673	1.0E-06

### 5. CLUV-induced proteomic changes

**Fig 6:**
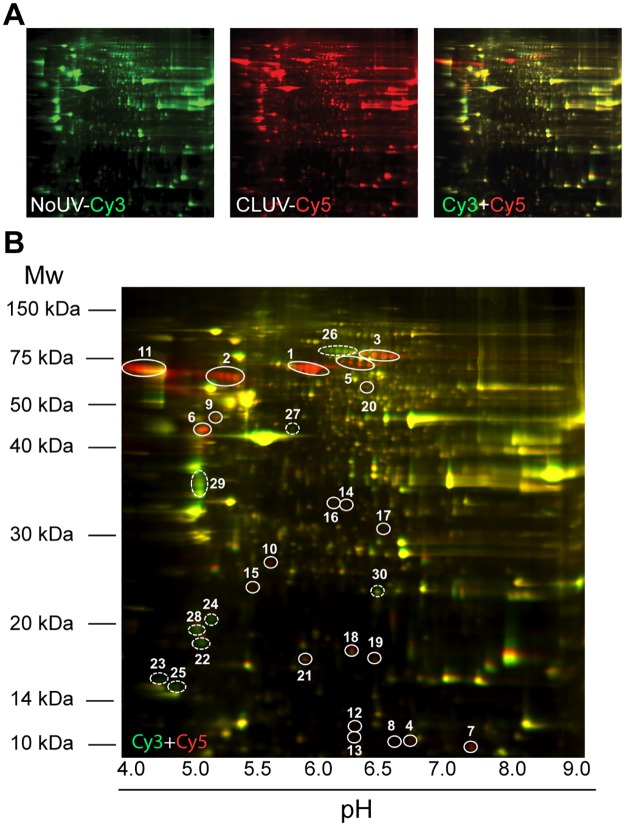
CLUV treatment induces proteomic changes. Three cell strains of NHDF subjected or not to a CLUV treatment and proteins were extracted. Proteins from the triplicate were pooled and proteome change was analyzed. **(A)** 2D-DIGE depicting protein expression differences between CLUV and untreated NHDF. The control (NoUV) was labeled with cy3 (left panel) and CLUV-treated cells (CLUV) with cy5 (middle panel). After labeling, proteins were separated on a 2D-DIGE according to their molecular weight and pH. Gels were merged (cy3/cy5) (right panel) to see proteomics changes. **(B)** Merged 2D-DIGE gel (cy3/cy5) depicting proteomic changes. Proteins with equal abundance between the CLUV-treated and the untreated NHDF are shown in yellow spot, while up-regulated proteins by the CLUV treatment appears in red and down-regulated in green. Full circles display up-regulated protein and dashed circles exhibit down-regulated proteins. A total of 30 proteins were further analyzed by mass spectrometry.

It is well documented that UV exposure leads to protein expression changes in skin cells [40]. More precisely, proteomic analysis reveals that a single acute UVB irradiation induces protein expression changes in skin fibroblasts [41, 42]. Since we discovered variations in CLUV-induced gene expression, and in accordance with previous studies observing the effect of UVB on proteome [41, 42], we sought to investigate proteomics changes in response to a CLUV treatment. A 2D-DIGE protein expression profiling analysis shows that the CLUV treatment induces proteomics changes (Fig 6). The 2D-DIGE/MS protein identification assay allows the identification of only a small fraction of the entire human proteome and most of the low-expressed proteins are not detected using this technique. Nonetheless, we have identified 2,500 proteins from which 30 were found deregulated by the CLUV irradiation. Indeed, we can observe some up-regulated and down-regulated protein induced by the CLUV pre-stimulation (Fig 6A, right panel). Spots with the highest expression were further analyzed (surrounded spots in Fig 6B) using Maldi TOF Mass spectrometry (S2 Table). Some redundancy in protein identification can be observed (#spot 3 and 5; #spot 6 and 9; #spot 23 and 25; and #spot 24 and 28), but these spots are close to each other and they are most likely artifacts from the spot identification.

Using the Reactome pathway database, we have identified biological process associated with the identified deregulated proteins (Table 2). While the gene expression profiling demonstrates a deregulation associated with stress response (Fig 5, Table 1), the proteome expression profiling emphasize on this process (Fig 6, Table 2). Indeed, we found 2 deregulated proteins in the programmed cell death process. However, this is in contradiction with our result depicted in Fig 4 showing that CLUV pre-stimulation does not influence UV-induced cell death sensitivity. On the other hand, the cell cycle progression delay induced by the CLUV treatment (Fig 3) is in accordance with the fact that we found protein deregulation in cell cycle process (Fig 6). Interestingly, we discovered an up-regulation of the DOT1 like histone lysine methyltransferase (DOT1L) (S2 Table), which is a protein involved in chromatin organization during DNA damage repair and the silencing of this protein exacerbate UV sensitivity [43]. We also found 5 of the 30 proteins modified by the CLUV treatment were involved in immune system biological process (Table 2 and S2 Table).

**Table 2:** List of biological processes of the 30 proteins analyzed.

Biological process	# protein	# proteins total	Protein name
Cell cycle	1	558	PSB9
Cell-cell communication	1	139	FLNA
Cellular responses to stress	1	354	SODM
Chromatin organization	1	226	DOT1L
Developmental biology	5	740	COF1—PSB9—ML12A - MYL9—MYL6
Disease	4	909	PSB9—ALBU—TRFL—CALR
DNA replication	1	106	PSB9
Extracellular matrix organization	1	272	CATD
Gene expression	1	1 346	PSB9
Hemostasis	7	517	HBD—EHD3—COF1—APOA1—ALBU—HBE—FLNA
Immune system	5	1 698	COF1—PSB9—CATD—TRFL—CALR
Metabolism	4	1 716	PSB9—APOA1—ALBU—HBA
Metabolism of protein	4	916	CATD—APOA1—TRFL—CALR
Muscle contraction	6	65	TPM1—VIME—CALD1—MYL6—MYL9—MYL12A
Programmed cell death	2	166	VIME—PSB9
Signal transduction	5	2 411	PSB9—APOA1- MYL9—MYL6—FLNA
Transmembrane transport of small molecule	2	595	ALBU—APOA1
Vesicle-mediated transport	4	463	HBA—ALBU—CALR—APOA1
Unidentified biolofical process	6	NA	DEST—STMN1—ZMYM4—NDRG1—NTAN1—CL055

## Discussion

UVB, a complete carcinogen, is the major factor involved in human skin cancer [44]. Cells react to UVB irradiation by triggering stress response at different molecular levels to protect themselves against this genotoxic stress [45, 46]. More precisely, cell cycle delay, DNA repair and apoptosis are amongst the most important protection mechanisms against UVB-induced skin cancer driver mutations [34, 47]. Nonetheless, even if these molecular mechanisms are well documented, most of previous studies were focused on the effect of single UVB irradiation [20, 48]. Even though repeated low dose of UVB are more representative of what humans are exposed to, only few studies have used this regimen [26, 49, 50]. Moreover, those previous studies have been conducted in different models (yeast, mice) and were mainly focused on the effect of CLUV alone, but not on the consequence of a CLUV pre-stimulation on a subsequent acute irradiation. In the present study, we used a CLUV irradiation protocol to mimic chronic irradiation and to understand how cells can cope with subsequent irradiation. We were aiming to determine whether the CLUV irradiation treatment would influence stress response mechanisms. To our knowledge, this is the first report studying the effect of a CLUV pre-stimulation on primary human diploid fibroblasts.

### 1. CLUV pre-stimulation enhance CPD repair

Using a slot-blot immunoassay, we measured the repair of UVB-induced CPD (Fig 2) after or not a CLUV pre-stimulation (Fig 1). Our results revealed that CPD repair is improved when cells are pre-stimulated with a CLUV regimen. This suggests an enhancement of the nucleotide excision repair (NER), the repair mechanism responsible for the CPD removal in human. Previous studies have demonstrated an improvement of DNA repair after a pre-stimulation with a chronic low dose of carcinogenesis, but none of them used CLUV irradiation [23, 24]. Although some studies have evaluated CLUV-induced CPD repair [26, 49], it has never been shown that a CLUV pre-stimulation enhance CPD repair. Indeed, none of the previous studies have investigated the effect of a CLUV treatment on DNA repair of newly formed CPD i.e. when cells are subsequently irradiated with an acute UVB dose.

We have also found that the CLUV treatment generates persistent CPD that remains on the DNA, at least 24 h post irradiation (Fig 2). It has been previously shown that some DNA regions are refractory to DNA repair [51, 52] and we suspect the CLUV-induced remaining CPD to accumulate in those regions. However, more investigation needs to be performed to determine the localization and implication of those residual CPD. Localization of those residual CPD might be important to determine their mutagenicity. Indeed, CPD can be induced in 4 types of dipyrimidine sites, i.e. TT, CC, CT and TC [53]. Since C→T transitions are the skin cancer causing mutations, T-containing CPD can be considered non-mutagenic [54]. If the residual CLUV-induced CPD are preferentially localized on T-containing dipyrimidine sites, the consequences on carcinogenesis is minimal. More work should be performed to determine the localization and implication of residual CPD.

### 2. Cell replication is delayed after a CLUV pre-stimulation

We have examined the effect of UVB irradiation (single UVB or CLUV) on cell replication and, as expected, we found that a single acute UVB dose is causing a delay in S-phase recovery when compared to un-irradiated cells (Fig 3). This delay is longer when cells are subjected to a CLUV treatment rather than a single dose (Fig 3). The delay in cell cycle for the single UVB irradiated cells could be explained by the DDR that halt cell cycle to allow efficient CPD repair [32, 55]. In CLUV treated cells, the longer delay might be the consequence of the residual CPD induced by the CLUV irradiation, which is consistent with previous study [56]. Indeed, it is well documented that DNA lesions are delaying DNA replication progression by blocking DNA polymerase [57]. When it happens, translesional DNA polymerase are needed to bypass the CPD [58, 59]. The translesion DNA synthesis (TLS) has been shown to be crucial for DNA damage tolerance [60] and also after a CLUV exposure [27]. TLS is much slower than the replication polymerases, mainly due to the lower processivity of TLS polymerase [60, 61]. We think this would explain, at least in part, the longer recovery time needed when cells are subjected to a CLUV irradiation.

### 3. CLUV pre-stimulation does not sensitize cells to UV-induced cell death

We investigated whether the CLUV pre-stimulation would influence cell sensitivity to UV-induced cell death. Surprisingly, our results revealed that the CLUV pre-treatment does not influence UV-induced cell death sensitivity (Fig 4). It has been previously shown that CPD are the principal UV-induced apoptosis inductor [17, 62, 63] and that unrepaired DNA damage could trigger cell death [64]. Since we found persistent residual CPD after the CLUV treatment (Fig 2), we were expecting to have a higher sensitivity to UV-induced apoptosis in CLUV pre-stimulated cells. However, the amount of CLUV-induced residual CPD is relatively minimal (Fig 2) compared to the amount generated by the UVB doses used to induce cell death (up to 40,000 J/m^2^) (Fig 4). This might explain why the CLUV pre-stimulation does not lead to higher UV-induced apoptosis sensitivity.

### 4. CLUV treatment induces transcriptomic and proteomic changes

Since we found that CLUV treatment had an influence on major stress response mechanisms, including cell cycle and DNA repair, we pursued the investigation by analyzing transcriptome and proteome changes induced by the CLUV. We first notice that CLUV pre-stimulation induces important changes in gene expression (Fig 5). It has been previously reported that CPD induce different transcriptional response associated to replication and DNA damage repair, suggesting that CPD are by themselves a cause of gene expression changes [65]. Since CLUV pre-stimulation leads to the accumulation of residual CPD (Fig 2), the observed gene expression changes induced by our CLUV treatment might be attributed to those residual CPD.

A further analysis of deregulated genes using categorization by gene ontology showed that 151 are directly related to cellular stress response, which represents 15.9% of all deregulated genes (Fig 5, Table 1). Despite the influence of cellular stress in general to gene expression [37], the UV rays are also known to induce transcriptomic changes [38]. Amongst the genes deregulated by a single UV irradiation, a large amount is associated with inflammation [37], senescence, cell cycle, DNA damage response and p53 signaling [66]. Noteworthy, all the studies done previously have been conducted using a single UV irradiation and we are the first one reporting the effect of chronic irradiation on human transcriptome. In our study, we have found that a CLUV treatment induces response to stress as described in previous study [66]. Indeed, our transcriptomic analysis found a deregulation in the ‘‘cell death”; “inflammatory response”; “response to wounding” and “response to stimulus” in the ‘‘response to stress” biological process. By example, we found that *XRCC2* gene 2.480 up deregulated. This protein is known to be crucial for DNA double strand break (DSB) repair by homologous recombination [67]. This result is in accordance with previous study where Garinis, G.A et al., demonstrate the role of unrepaired CPD in UV-induced DNA breaks [65]. Furthermore, *IL-33* and *CRH* gene, both important in the inflammatory response are deregulated (S1 Table). IL-33 plays a role in the activation of innate immune system [68] and CRH is a major coordinator of the stress response. Previous studies pointed out the key role of CRH in skin response to stress [16].

We did not find any difference in NER-related gene expression, which was expected since the regulation of NER efficiency is mainly driven at the post-translational level rather than the transcriptional level.

Since our results showed that CLUV irradiation affects gene expression, we further analyzed whether the CLUV treatment affects protein levels (Fig 6). The large-scale study of protein revealed 2 500 potential proteins on the 2D-gel, from which 30 proteins were at least 2 times deregulated (Fig 6B). Those 30 proteins were identified by mass spectrometry, 21 are up-regulated and 9 down-regulated by the CLUV pre-stimulation (S2 Table). We found the antioxidant enzyme Superoxide dismutase mitochondrial (SODM) to be down-regulated (S2 Table). In addition to protect against oxidative stress, SODM is known to confer resistance to apoptosis [69]. Furthermore, previous work has demonstrated that UVB induces proteomics changes, especially an up-regulation of vimentin [42]. This has been suggested to contribute to cells resistance to UVB-induced damage. Vimentin is also found to be up-regulated in our present work (Table 2 and S2 Table), and is known as a contributor of apoptosis [70], which is in contradiction with our data showing that CLUV pre-stimulation does not influence UV-induced cell death sensitivity (Fig 4). In fact, we could see a higher sensitivity to UV-induced cell death at 40,000 J/m^2^, but it was not significant. More work would need to be done to determine the exact influence of vimentin upregulation and SODM down-regulation in UV-induced cell stress response post-CLUV irradiation.

Furthermore, the up-regulation of DOT1L was of interest since its role in chromatin structure to regulate DNA damage response is well established [71]. In addition to be crucial in chromatin organization, recent study demonstrates its critical role on cell cycle regulation [72] but also on UV sensitivity [43]. Furthermore, DOT1L plays a role in DSB repair [73], which is in accordance with our transcriptomic results (S1 Table). Additional analysis should be performed to clarify the role of those proteins in cellular response to a CLUV pre-stimulation.

Finally, 5 of the 30 proteins found in our analysis are implicated in the immune system process (Table 2). This is in accordance with previous report showing the involvement of UV exposure in immune system changes [74]. However, further investigation is needed to correlate the effect of CLUV treatment on the immune system.

To our knowledge, this study represents the first demonstration that a chronic irradiation can influence genotoxic stress response. Indeed, using 4 different strains of NHDF, we have shown that cells can respond to chronic UV irradiation by adapting their genotoxic stress response mechanisms, i.e. cell cycle and DNA repair. This cellular adaptability might reflect the potential human skin adaptation to chronic exposure to sunlight. However, the transcriptome and proteome analysis have shown that those stress response mechanisms are not the only ones affected by the CLUV treatment and further analysis need to be done to shed light on those mechanisms and their consequence for cells.

## Materials and methods

### Ethic statement

All experiments performed in this study were conducted in accordance with our institution's guidelines and the Declaration of Helsinki. The research protocols received approval by the Centre de Recherche du CHU de Québec (CRCHUQ) institutional committee for the protection of human subjects.

### Cell culture

NHDF are from human skin biopsies (mastectomy) of 4 healthy women from 18 to 38 years old. Cells were used between passage 11 and 13 [75]. They were cultured in Dulbecco’s modified Eagle’s Medium (DMEM) (Corning cellgro, VA, USA) complemented with 10% FBS and 1% penicillin/streptomycin (Wisent, QC, CA) at 37°C, 5% CO_2_.

### UVB irradiation and CLUV treatment

NHDF were irradiated using RPR-3000 UVB lamps (Southern New England Ultraviolet Co.) with an emission peak of 300 nm [76]. A cellulose acetate sheet (Kodacel TA-407, clear 0.015 in.; Eastman-Kodak Co.) was used to filter out wavelength below 295 nm [76]. The acute UVB irradiation of 400 J/m^2^ has been chosen according previous studies [31, 77] and corresponds to 5–10 min of solar exposure at zenith sun [78]. The UVB dose chosen for the chronic irradiation (CLUV) has been selected based on previous experiments and cell sensitivity [79]. Each irradiation in of the chronic protocol corresponds to around 1 min of direct solar exposure.

#### Irradiation protocol

Fig 1 depicts the irradiation protocol.

#### CLUV

Confluent NHDF were first exposed to a CLUV treatment consisting of 75 J/m^2^ of UVB (100 J/m^2^ for the gene profiling experiment, Fig 5) every 12 h for 7.5 days. Each day, cells then received a total of 150 J/m^2^ of UVB (200 J/m^2^ for the gene profiling experiment, Fig 5). After 7.5 days, cells have received a total of 15 irradiations (Fig 1.2 and 1.3). After the CLUV treatment, cells were incubated for 12 h and were either harvested (corresponding to the 0 h time point) or 24 h later (Fig 1.2). Irradiation was performed in PBS to avoid cell dehydration and oxidative stress (Corning cellgro). The DMEM medium was filtered and reuse between irradiations.

#### Acute

Confluent NHDF were exposed to a single acute UVB irradiation of 400J/m^2^ (200 J/m^2^ for the cell cycle experiment, Fig 3). Prior to the single acute UVB irradiation, medium was filtered and replace with PBS at the same frequency (every 12 h, for 7.5 days) as the CLUV treated cells in order to mimic experimental stress.

#### CLUV+Acute

CLUV treated cells were irradiated with 400 J/m^2^ UVB 12 h after the last irradiation of the CLUV treatment.

### DNA damage and repair

#### DNA extraction

Total DNA was extracted using a DNeasy Blood and Tissue Kit (QIAGEN) following the manufacturer’s protocol with an additional RNase treatment. DNA concentration was determined using a spectrophometer (NanoDrop 2000; Thermoscientific).

#### DNA slot blots

The immuno-slot-blot technique was performed as previously described [52]. Briefly, after alkaline DNA denaturation (10 min 56°C, followed by 3N of NaOH), DNA was blotted on positively charged nitrocellulose membranes (Bio-Dot^®^ SF Microfiltration Apparatus), and DNA was heat fixed to the membrane (80°C, 3 h). Membranes were then blocked with 5% nonfat dry milk and hybridized with a mouse anti-CPD monoclonal antibody (Cosmo Bio Co., clone TDM-2) diluted 1:5 000 in 1% milk + 0.05% tween. The secondary HRP-conjugated antibody (Rabbit anti-mouse) (Jackson ImmunoResearch) was diluted 1:5 000 in 1% milk + 0.05% tween. A mouse anti-ssDNA monoclonal antibody (EMD Millipore, clone 16–19) diluted 1:1 000 was used to detect DNA. Membranes analysis and quantification were performed with C-DiGit^®^ Blot Scanner (LI-COR Biosciences).

NHDF from 3 different cell strains were used for this experiment, and the slot blot was performed at least twice for each NHDF strain cells. *P*-values were derived from the two-tailed heteroscedastic Student’s t-test.

### Cell cycle analysis by flow cytometry

NHDF cells were subjected to either an acute dose (acute), a CLUV treatment (CLUV) or un-irradiated (NoUV). Cells were then re-seeded at a density of 8.3x10^3^ cells/cm^2^ and incubated for different time points (0 to 36 h). They were then fixed with ethanol 70% and stained using propidium iodide (PI). Cell cycle distribution was analyzed by flow cytometry. Four NHDF cell strains were used for this experiment.

### UVB-induced cell death assay

Confluent NHDF pre-stimulated or not by the CLUV treatment were subjected to acute UVB dose, ranging from 0 to 40,000 J/m^2^. Sixteen h after acute irradiation, cells were harvested and stained with Annexin V/PI apoptosis kit (Molecular probes, Eugene, OR) as previously described [31, 75]. Briefly, 16 h after acute UVB dose, cells were harvested and resuspended in Annexin V binding buffer. The staining annexin V and PI are added and cells are incubated 15 min at room temperature. Analysis of apoptotic (Annexin V positive cells) and necrotic (PI positive cells) was performed by flow cytometry. Four different NHDF cell strains were used and *p*-values were derived from the two-tailed heteroscedastic Student’s *t*-test.

### Gene expression profiling analysis

#### RNA isolation

Total RNA was isolated from CLUV pre-stimulated and un-irradiated controls (NoUV) using TRIzol^®^ Reagent (Life Technologies) according to manufacturer’s instructions. RNA quantity and quality were assessed using a 2100 Bioanalyzer Instruments (Agilent Technologies) according to the manufacturer’s protocol.

#### Sample preparation and procedure

Sample preparation was performed following to One-color Microarray-Based Gene Expression Analysis protocol and as described in [80].

#### Microarray hybridization and analysis

150 ng of amplified cRNA was incubated on a G4851A SurePrint G3 Human Ge 8 x 60 K array slide (Agilent Technologies). Slides have over 60 000 targets of the global human genome. After 18 h of slide’s hybridization, they were washed and scanned on an Agilent SureScan Scanner.

#### Data analysis

A data report was produced by Arraystar v 4.1 software (DNASTAR), which includes the scatter plot and heat map of deregulated genes. Further statistical analyses were performed in collaboration with the Bioinformatics Platform of the CHU de Québec. GO enrichment analyses were done on the list of variant genes (fold-change ≥ 2) using the "biological process" ontology. Enrichment analyses were performed using BiNGO (v 3.0.3) on the Cytoscape software platform (v 3.2.1) [81, 82]. Enrichment was tested by hypergeometric test (without replacement) with Bonferroni correction for multiple testing, and annotations are said to be significantly enriched at corrected p-value ≤ 0.01.

### Proteome expression analysis

#### Sample preparation and procedure

Three cell strains of CLUV treated NHDF were pooled together. The same 3 cell strains un-irradiated (NoUV) were also pooled and were used as baseline. Proteins were extracted using RIPA buffer (Thermo scientific) and protein concentration was determined by the BCA assay using Bio-Rad protein kit.

#### Two-Dimensional Difference Gel Electrophoresis (2D-DIGE)

The two-dimensional difference gel electrophoresis (2D-DIGE) and mass spectrometry analysis were performed by Applied Biomics (Hayward, CA). Before proceeding with the 2D-DIGE, a 5 kDa MWCO spin column was used to replace RIPA buffer with the 2D lysis buffer (7M urea, 2M thiourea, 4% CHAPS, 30 mM Tris-HCl). Procedure of 2D-DIGE has been performed as previously described [83]. Briefly, samples were first labeled with CyDye DIGE fluors (NoUV: Cy3; CLUV: Cy5). Then, for the first dimension, the isoelectric focusing (IEF) has been used to separate both samples, and then a SDS polyacrylamide gel electrophoresis (SDS-PAGE) has been used for the second dimension. Image acquisition is performed with Typhoon image scanner and analysis of scan was performed using ImageQuant software. Using DeCyder analysis software, the protein levels were determined. The DeCyder software found the 70 most deregulated proteins. Only proteins deregulated at least 1.5 times were sent for mass spectrometry analysis.

#### Data analysis

MS analysis is based on peptide fingerprint mass mapping. MASCOT software was used to identify proteins according to their peptide fingerprint. Proteins were accepted on the basis of peptide count and total ion confidence interval (C.I. %) (S2 Table). Protein identifications were considered accurate if there was at least 3 peptides match and if total ion C.I. % was greater than 95,0% when calculated from MS data.

Proteins functional analysis was performed using the Reactome pathway knowledge base (Reactome v54), an open-source, open-data and peer-reviewed database of human pathways and reactions [84, 85].

## Supporting information

S1 Table Compilation of deregulated genes related to stress response. (TIF)

S2 Table List of the 30 proteins deregulated and their mass spectrometry characteristics. (TIF)
