# Supplementary figures and images for: Influence of a pre-stimulation with chronic low-dose UVB on stress response mechanisms in human skin fibroblasts
Source: PLoS One. 2017 Mar 16;12(3):e0173740. doi: 10.1371/journal.pone.0173740 (PMC5354420; doi:10.1371/journal.pone.0173740)

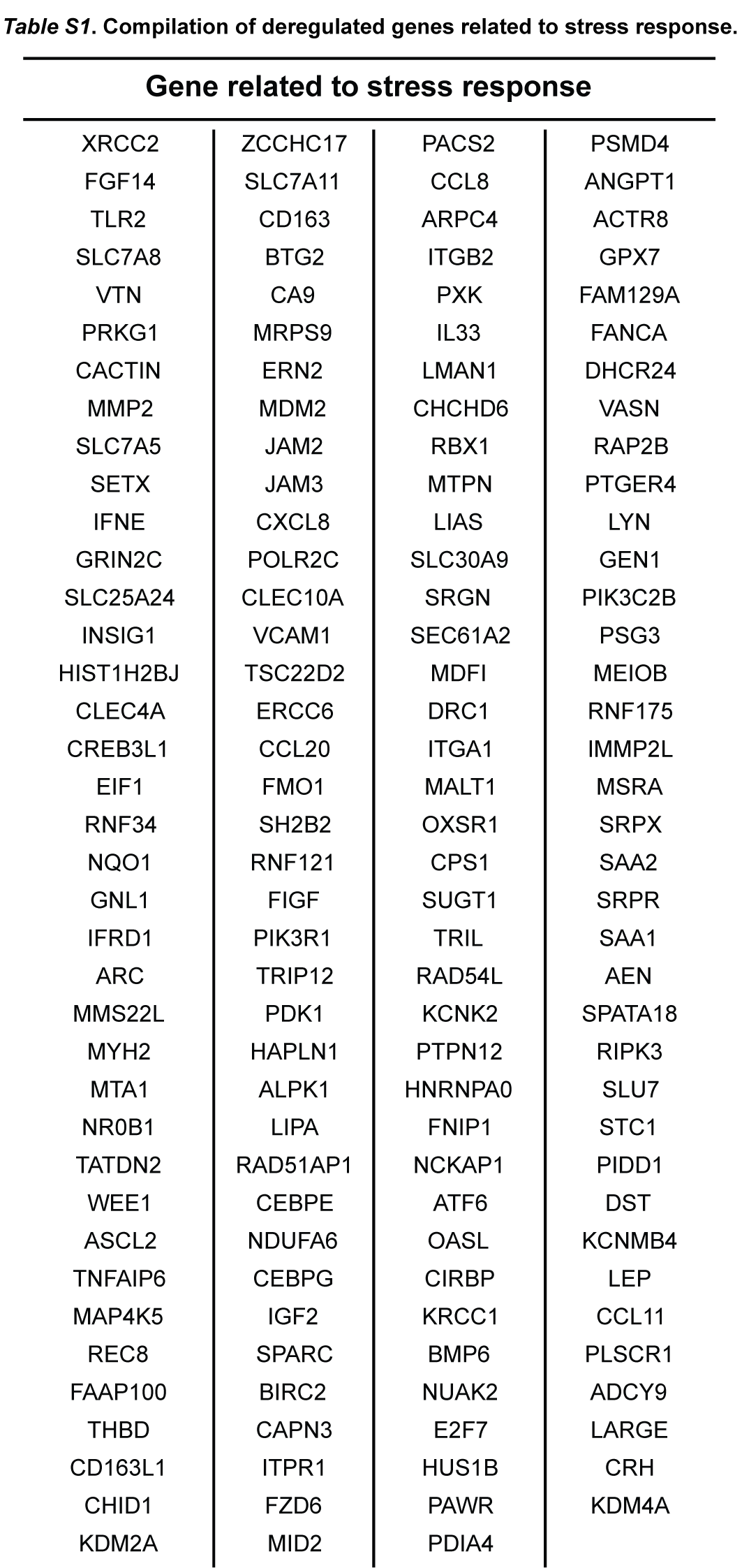

Supplement: S1 Table — (TIF) [file pone.0173740.s001.tif]

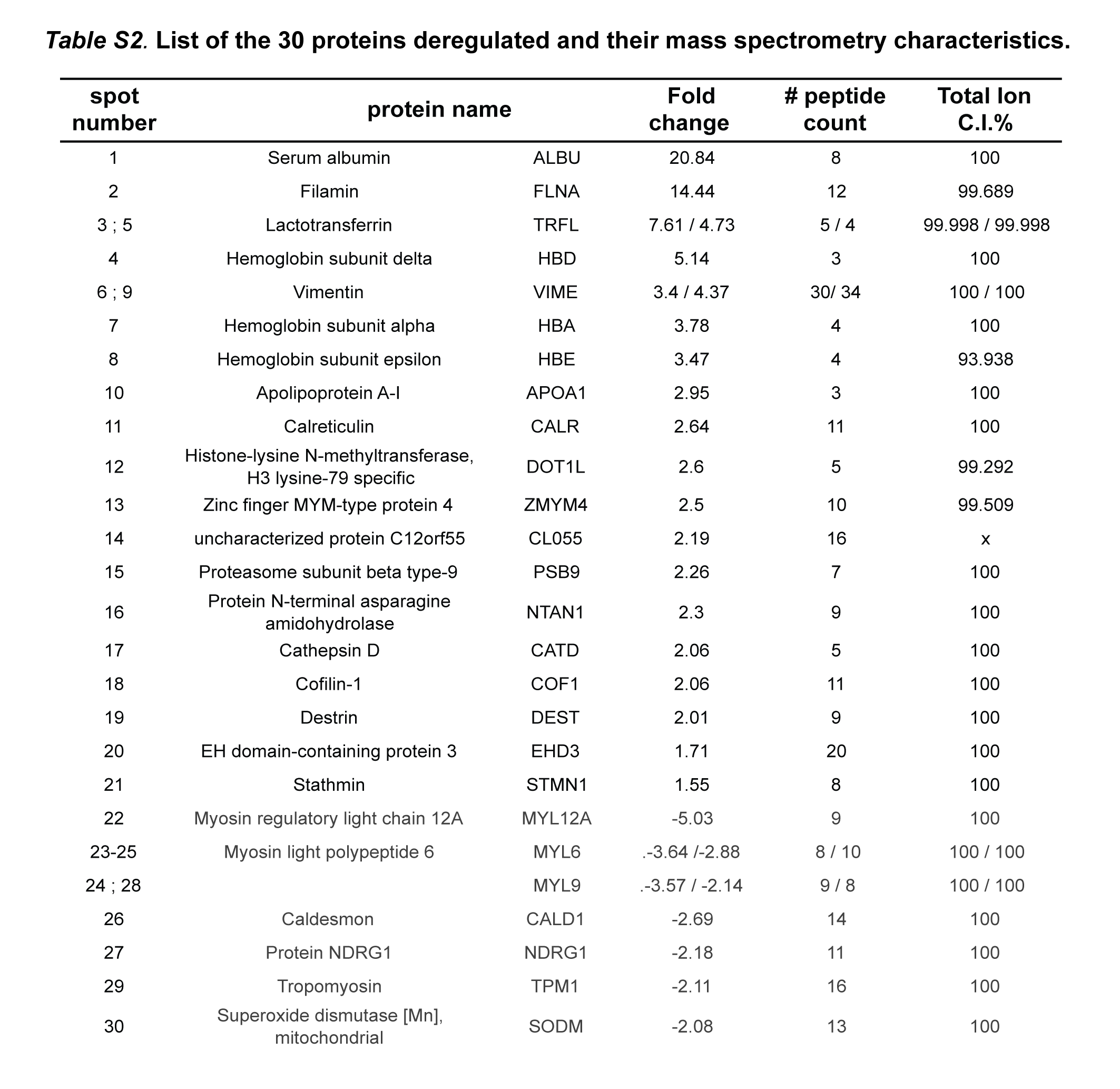

Supplement: S2 Table — (TIF) [file pone.0173740.s002.tif]
